# FTIR and Py–GC–MS data of wood from various living oak species and Iberian shipwrecks

**DOI:** 10.1016/j.dib.2018.11.032

**Published:** 2018-11-10

**Authors:** Mohamed Traoré, Joeri Kaal, Antonio Martínez Cortizas

**Affiliations:** a Ciencia do Sistema Terra, Departamento de Edafoloxía e Química Agrícola, Universidade de Santiago de Compostela, Campus Sur s/n, Santiago de Compostela 15782, Spain; b Instituto de Ciencias del Patrimonio (Incipit), Consejo Superior de Investigaciones Científicas (CSIC), s/n, A, Av. de Vigo, 15705 Santiago de Compostela, A Coruña, Spain

## Abstract

Data in this article are related to the chemical characterization of various oak wood samples. Data have been obtained by the application of Fourier Transform Infrared (FTIR) spectroscopy and pyrolysis-gas chromatography-mass spectrometry (Py–GC–MS) to living tree species and shipwreck wood fragments. Measurements were performed on individual rings in order to facilitate the understanding of the variability in wood chemical composition along the radial cores, i.e. the same kind of material traditionally used for dendrochronological analysis. The data in this article is labelled according to the anatomical sections of the wood (sapwood, transition wood and heartwood) where the samples were taken. The experimental background and the results can be found in the related research article, “Chemometric tools for identification of wood from different oak species and their potential for provenancing of Iberian shipwrecks (16th–18th centuries CE)” (Traoré et al., 2018).

**Specifications table**

**Table:** 

Subject area	*Geochemistry, Forestry, Environmental science*
More specific subject area	*Organic geochemistry, Spectroscopy, Environmental chemistry*
Type of data	Spreadsheet files (Microsoft Excel, *.xls)
How data was acquired	*The FTIR-ATR data were recorded on a Cary 630 (Agilent Technologies). Py–GC–MS data were acquired using a CDS Pyroprobe 5000 (for pyrolysis at 650 °C for 10 s) coupled to an Agilent 6890 gas chromatograph and 5975B mass selective detector.*
Data format	*Relative abundances data.*
Experimental factors	*Living tree wood cores from four oak wood species (Quercus faginea, Quercus petraea, Quercus pyrenaica and Quercus robur) and wood fragments from four shipwrecks (Belinho, Magdalena, Ribadeo and Yarmouth).*
Experimental features	*Living tree and archaeological wood samples were simply conditioned for the analysis. More than thousand FTIR spectra and about 305 Py–GC–MS measurement were recorded and results were analysed and interpreted by the mean of Principal Component Analysis and Discriminant Analysis.*
Data source location	*Basque Country, Cantabria, Galicia (Spain)*
	*Belinho (Portugal)*
	*The Isle of Wight, (United Kingdom).*
Data accessibility	*Data provided in the article are publicly available on Mendeley*
	Data: https://data.mendeley.com/datasets/528y6ckjrc/draft?a=9903b506-6ff9-47fe-ad04-bffba5f075ba
Related research article	*M. Traoré, J. Kaal and A. Martínez Cortizas, “Chemometric tools for identification of wood from different oak species and their potential for provenancing of Iberian shipwrecks (16th-18th centuries AD)”, Journal of Archaeological Science*100 (2018) 62–73 [1]

**Value of the data**

• The FTIR and Py–GC–MS data in this paper refer to wood chemical composition through the molecular characteristic.
• Data in this paper provide valuable wood chemical detail related to differences between sapwood, transition wood and heartwood; between various wood species; and also between sound wood and underwater archaeological wood.
• This data are useful for researchers in order to extend the statistical analyses.

## 1 Data

Data presented here include FTIR and Py–GC–MS values of living tree wood and shipwreck wood samples, from four oak (*Quercus* spp.) species. Samples were collected in different forests in the Cantabrian and the Basque country regions in Spain. Unknown species of oak wood fragments were collected from various shipwrecks with Iberian shipbuilding features.

Data are subdivided in three excel files. The first excel file (relative intensities in the fingerprint region) contains the relative FTIR-ATR intensities of the main absorption bands of the fingerprint region (1800-800 cm^−1^) for sound and shipwreck woods. Band selections were carried out by considering only FTIR peaks related to the main wood chemical constituents (polysaccharide and lignin). The second excel file (relative abundances of pyrolysis products) contains the relative abundances of pyrolysis products for sound and shipwreck woods, expressed as relative proportions of total peak area; again, focused on polysaccharide and lignin compounds, in addition to several minor products. The third file (extended heartwood spectral dataset) contains the extended heartwood spectral dataset (see article [1]) of the main absorption bands of the fingerprint region for sound woods. The main reason for the extension of the dataset was to increase the consistency of the discriminant models, taking advantage of the fact that FTIR is a rapid and economical technique (the extended dataset was not created for Py–GC–MS). The extended dataset used for the discriminant analysis consists of a training set and a validation set.

## 2 Experimental design, materials, and methods

Fourier Transform Infrared spectroscopy (FTIR) and pyrolysis-gas chromatography-mass spectrometry (Py–GC–MS) are the two analytical techniques applied for wood chemical characterization in this article. More details about the analytical techniques can be found in [2] and [3].Wood cores were retrieved at breast height using an increment borer in different sampling sites. Furthermore, wood fragments were collected from a series of shipwrecks with Iberian shipbuilding features. No chemical treatment was applied to the analysed wood samples (living tree wood cores and fragments from shipwreck), with the exception of drying at 30 °C for two weeks. The shipwreck woods were cleaned by removing sediment and shipworm shells. For all wood samples, the surfaces were clean-cut to better visualize growth rings. Measurements were taken on individual rings aiming to improve data resolution. In order to increase the consistency of the discriminant models (for species differentiation), additional spectra from individual rings belonging to the heartwood of living trees wood cores (“extended heartwood dataset”) were recorded. Quality assessment of the FTIR and Py–GC–MS were evaluated according to protocols provided in earlier publications [2], [3]. FTIR measurements were performed throughout the wood cores at 1 cm intervals. In total, >1000 FTIR spectra were recorded for this research. Peak absorption intensities were used to calculate relative intensity at related FTIR bands; the relative intensity permits to get comparable result for all the collected spectra without any particular interference related to individual tree rings. Since it is a more time- and effort-demanding technique, Py–GC–MS was performed on eight to ten individual rings throughout the cross sections. As such, a total of 305 Py–GC–MS chromatograms were obtained for wood samples of similar size (<1 mg). Peak areas were used to calculate relative proportions of the pyrolysis products. The application of principal component analysis allowed the identification of the mechanisms that control the variability in the pyrolysis fingerprints of the wood. Finally, discriminant analyses were applied to predict the group membership of the samples, according to the discriminant models.
