# Supplementary material for: FTIR and Py–GC–MS data of wood from various living oak species and Iberian shipwrecks
Source: Data Brief. 2018 Nov 10;21:1861–3. doi: 10.1016/j.dib.2018.11.032 (PMC6260304; doi:10.1016/j.dib.2018.11.032)
Supplement: Supplementary file 1 — Transparency document [file mmc1.docx]

Conflict of Interest and Authorship Conformation Form

- All authors have participated in (a) conception and design, or analysis and interpretation of the data; (b) drafting the article or revising it critically for important intellectual content; and (c) approval of the final version.
- This manuscript has not been submitted to, nor is under review at, another journal or other publishing venue.
- The authors have no affiliation with any organization with a direct or indirect financial interest in the subject matter discussed in the manuscript
- The following authors have affiliations with organizations with direct or indirect financial interest in the subject matter discussed in the manuscript:

Author’s name Affiliation

Mohamed Traoré Universidade de Santiago de Compostela

Joeri Kaal Universidade de Santiago de Compostela

Antonio Martínez Cortizas Universidade de Santiago de Compostela
